# Donor-Recipient Mismatch in Lung Transplantation: The Role of Graft Sizing in Clinical Outcomes

**DOI:** 10.3389/ti.2025.14387

**Published:** 2025-04-09

**Authors:** Chiara Catelli, Miriana D’Alessandro, Andrea Lloret Madrid, Antonella Fossi, Federico Franchi, David Bennett, Piero Paladini, Elena Bargagli, Luca Luzzi

**Affiliations:** ^1^ Lung Transplant Unit, Azienda Ospedaliero-Universitaria Senese, Siena, Italy; ^2^ Department of Medicine, Surgery and Neurosciences, University of Siena, Siena, Italy; ^3^ Respiratory Diseases Unit, Azienda Ospedaliero-Universitaria Senese, Siena, Italy; ^4^ Cardiothoracic and Vascular Anesthesia and Intensive Care Unit, Azienda Ospedaliero-Universitaria Senese, Siena, Italy; ^5^ Thoracic Surgery Unit, Azienda Ospedaliero-Universitaria Senese, Siena, Italy

**Keywords:** lung transplantation, lung resection, donor selection, size matching, allograft

## Abstract

Lung transplantation is a life-saving procedure for end-stage lung diseases. Size matching is critical in the donor-recipient selection process. This retrospective study analyzed 146 patients who underwent lung transplantation between 2013 and 2023. Patients who required graft resizing were assigned to the sizing group (S), non-resizing cases to the non-sizing group (NS). The primary goal was to identify predictive factors for graft resizing. Secondary endpoints included ischemia time, ventilation time, primary graft dysfunction (PGD) and hospital stay. The S group was further stratified on baseline parameters to assess differences in outcomes. Recipient height and single transplants were higher in the NS group. Donor-recipient height ratio was the only predictor for resizing (p = 0.02). Postoperative outcomes and overall survival were similar between the groups. In Group S, male patients showed higher rates of acute kidney injury (AKI) and chronic rejection, the former being associated also with anatomical resections; patients older than 50 experienced higher rates of PGD. Graft resizing is a feasible strategy for addressing size mismatch, but it is associated with increased risks of PGD and AKI, particularly in older male recipients and those undergoing anatomical resections. These findings highlight the importance of careful preoperative donor-recipient size matching.

## Graphical Abstract

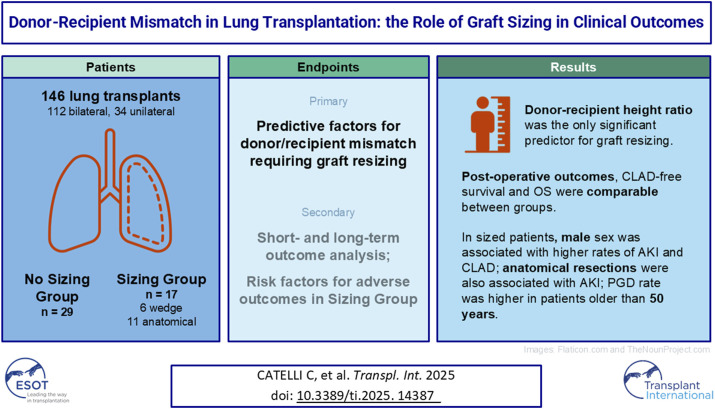

## Introduction

Despite its success in prolonging survival, lung transplantation faces several challenges, one of the most significant being the mismatch between the donor’s and recipient’s lung size and physiological characteristics. Such mismatches can contribute to a range of postoperative complications, including primary graft dysfunction, bronchiolitis obliterans syndrome, and overall reduced graft survival [1, 2]. Several factors—both anatomical and physiological—contribute to this mismatch, including the recipient’s chest wall mechanics, lung compliance, and the size of the donor’s lungs relative to the recipient’s thoracic cavity [3].

In particular, the recipient’s lung capacity and thoracic dimensions can vary significantly, creating potential challenges when selecting an appropriate donor lung [4, 5]. Over- and under-sizing of the lung graft are associated with various complications, ranging from impaired gas exchange to increased risk of rejection and graft dysfunction [6]. Conversely, an undersized graft may fail to meet the recipient’s functional needs, compromising postoperative outcomes and leading to complications such as early graft failure.

In response to these challenges, there has been a growing focus on developing strategies to optimize the donor-recipient match [7, 8]. Graft sizing techniques have emerged as a potential solution, utilizing advanced imaging methods, such as three-dimensional computed tomography (CT) volumetry [9, 10], to better assess the donor lung’s size and its compatibility with the recipient. However, in some cases, particularly when the recipient is in poor general condition or has a rare blood type, oversized organs may be necessary.

In cases where there is a small size discrepancy between the donor and recipient, limited non-anatomic or sublobar graft resections are often effective. However, for more significant mismatches, lobar reduction is typically the preferred surgical approach [11]. Due to the technical challenges, the available case series on this technique are few [12], and the outcomes reported across studies have been inconsistent.

This study aims to investigate the key predictive factors that contribute to mismatch between donor and recipient in lung transplantation and the subsequent need for graft sizing. Furthermore, it will evaluate the clinical outcomes of patients undergoing graft sizing procedures, analyzing potential risk factors for poorer outcomes in certain patient categories.

## Materials and Methods

### Patients

This is a retrospective study involving all patients who underwent single or double lung transplantation between 1 January 2013, and 31 December 2023, at the Lung Transplant Unit of the University Hospital of Siena. The study was approved by the Institutional Review Board (IRB). Patients who underwent graft reduction for reasons other than mismatch (such as pulmonary contusions, lobar edema, or parenchymal consolidation) were excluded from the study, as these lungs may have been compromised before transplantation, thus increasing the risk of complications regardless the sizing procedure. The type of surgical resection performed in our study was operator-dependent and based primarily on four assessments. These included [1]: a reduction in systemic pressure due to heart compression observed during chest closure [2]; an evident mismatch identified either before the graft implantation or during chest closure [3]; atelectasis of part of the lung parenchyma during recruitment due to insufficient thoracic cavity size and [4] an increase in registered ventilatory pressures observed during chest closure.

Patients were divided into two groups based on the need for sizing at the time of implantation due to dimensional mismatch between the donor and recipient. The sizing group (Group S) included patients who underwent atypical and/or anatomical lung resections (segmentectomy, lobectomy), while Group NS included all patients who did not require graft resizing. At our center, donor lungs are allocated based on blood group, Lung Allocation Score (LAS), height, and age.

In the two groups, discrepancies between the donor and recipient were analyzed in terms of sex, race, BMI ratio (donor/recipient), height ratio (donor/recipient), weight ratio (donor/recipient), and age ratio (donor/recipient). The comorbidities of both the recipient and donor, as well as the type of transplant performed (single or double), were also analyzed and compared. The primary endpoint of the study was to evaluate which characteristics of the donor and recipient were predictive factors for D/R mismatch requiring lung resection on the graft. The secondary endpoint was to analyze primary outcomes, such as overall survival, and secondary outcomes, such as the occurrence of Primary Graft Dysfunction (PGD), Chronic Lung Allograft Dysfunction (CLAD), ischemia time, and duration of mechanical ventilation in the two groups. In patients who received sized grafts, outcomes were then stratified based on the following patient characteristics: BMI (greater or less than 25); sex (male or female); age (greater or less than 50 years); type of end-stage pulmonary disease (restrictive or obstructive); type of sizing (lobectomy/segmentectomy or atypical resection). Ethical approval was not required for the study, in accordance to the local legislation, because of its retrospective nature.

### Surgical Procedure

The lung transplant procedure was standard, performed through a clamshell incision for bilateral transplants or a posterolateral thoracotomy for single transplants. In the case of graft sizing, anatomical resections were performed at the back table or after lung implantation, before hemostasis and chest closure, using mechanical staplers for bronchial and vascular structures, while atypical resections were always performed after lung implantation with the use of mechanical staplers. The decision to perform graft reduction and determine the type of resection is based on visual inspection and clinical parameters. Specifically, the final decision on the need for sizing is made after a thorough inspection of the recipient’s thoracic cavity. If the mismatch is immediately apparent, sizing is performed through anatomical resection before graft implantation. If, however, the mismatch is not evident during clinical inspection but hemodynamic instability occurs during chest closure due to compression of the overinflated lung on the cardiac cavities, resection is performed at the end of the procedure. The choice of which part of the lung to sacrifice was based on the recipient’s thoracic configuration, with middle lobectomy or lingulectomy being preferred in cases of antero-posterior mismatch, while the sacrifice of the lower lobes was preferred in cases of diaphragmatic elevation [13]. The decision was also influenced by the appearance of the lung, such as sacrificing the most difficult-to-recruit or edematous portion after implantation. Postoperatively, patients received appropriate antibiotic prophylaxis (Vancomycin, Cefepime, Ganciclovir, and Ig-CMV) and immunosuppressive therapy (Basiliximab, methylprednisolone, tacrolimus, and mycophenolate mofetil).

### Statistical Analysis

The results data were expressed as mean ± standard deviation and median (interquartile range), as appropriate. Non-parametric tests were adopted for data analysis: comparisons between two groups were determined by Mann-Whitney U test; ANOVA test (Kruskal–Wallis and Dunn’s multiple tests) were performed to compare more than two groups. Contingency analysis was performed to evaluate the association and the independence between the parameters as well as to calculate various association measures. Correlations between variables were determined by Spearman correlation coefficient. Survival distribution in the two groups was evaluated using a weighted Kaplan–Meier approach. Statistical analysis was performed by GraphPad Prism 9.10.3, XLSTAT 2021 and Jamovi software.

## Results

The study included 146 patients who underwent lung transplantation at our center. 17 patients (11.6%) underwent lung resection due to graft mismatch (Sizing Group or Group S), while the remaining 129 did not require lung resection (Non-Sizing Group or Group NS). One patient was excluded from the study as they underwent graft resection due to pulmonary consolidation in the right lower lobe, which developed during graft reperfusion via *ex vivo* lung perfusion (EVLP). Table 1 summarizes the recipient characteristics, stratified by group. In the Sizing Group, the average age of the recipients was 55 years (range 23–64), with 53% (9 patients) being female and an average BMI of 26.4, indicating mild overweight. The majority of patients in Group S had a restrictive type of end-stage lung disease (8 patients, 47%).

**TABLE 1:** Recipients’ characteristics.

Variable	Group NS N = 129	Group S N = 17	P
Age (y)	55.5 (17–66)	55.0 (23–64)	0.462
Sex (M; F)	84 (65%); 45 (35%)	8 (47%); 9 (53%)	0.147
BMI	24.0 (14.8–34.2)	26.4 (17.0–34.9)	0.357
Height (cm)	169 (150–196)	164 (150–178)	**0.004**
Weight (kg)	68 (38–120)	70 (40–101)	0.852
LAS	20 (2–63)	31 (4–88)	0.668
FEV1%	38 (9–125)	35 (12–90)	0.975
FVC%	49 (20–168)	51 (15–87)	0.741
DLCO%	29 (2–62)	30 (5–85)	0.475
Pattern of lung disease			0.944
Restrictive	52 (40.3%)	8 (47.1%)	
Obstructive	8 (6.2%)	3 (17.6%)	
Cystic Fibrosis	23 (17.8%)	3 (17.6%)	
Mixed	25 (19.4%)	3 (17.6%)	
Transplant type			**0.020**
SLTX	31 (24.0%)	3 (17.7%)	
BLTX	98 (76.0%)	14 (82.3%)	
Comorbidities			
Arterial hypertension	37 (29%)	4 (24%)	0.657
Pulmonary hypertension	19 (15%)	3 (18%)	0.762
Dyslipidemia	18 (14%)	2 (12%)	0.796
Diabetes mellitus	22 (17%)	4 (24%)	0.522
Obesity	8 (6%)	1 (6%)	0.953
Osteopenia/osteoporosis	47 (36%)	7 (41%)	0.721

The categorical variables are presented as percentages; the continuous variables are expressed as the median and interquartile range (IQR). BLTX, Bilateral Lung Transplant; BMI, Body Mass Index; DLCO, Diffusing Capacity of the Lung for Carbon Monoxide; FEV1, Forced Expiratory Volume in one second; FVC, Forced Vital Capacity; LAS, Lung Allocation Score; SLTX, Single Lung Transplant. Significant p values are reported in bold type.

No statistically significant differences were observed between the groups in terms of age at transplant, sex, or BMI. However, a statistically significant difference in recipient height was observed (164 cm in Group S vs. 169 cm in Group NS, p = 0.004), with shorter recipients in the Sizing Group. No significant differences were found in preoperative forced expiratory volume in one second (FEV1%), forced vital capacity (FVC%), or diffusing capacity of the lung for carbon monoxide (DLCO%), nor in the type of end-stage lung disease between the two groups. Bilateral lung transplants were more frequent in Group S (82.3%) compared to Group NS (76.0%, p = 0.02). No significant differences were observed between the two groups in terms of patient comorbidities. Despite this, it was noted that the Lung Allocation Score (LAS) was higher in Group S (31 vs. 20), which is clinically relevant, although not statistically significant (p = 0.668).

Table 2 presents the characteristics of the donors and the donor-recipient discrepancies. No differences were observed in the comorbidities of the donors between the two study groups, nor in the frequency of smoking habits. The ratio between the donor’s and recipient’s age was lower in Group NS (0.845) compared to Group S (0.990, p = 0.041). A statistically significant difference was also observed in the ratio between the donor’s and recipient’s height (1.01 in Group NS vs. 1.04 in Group S, p = 0.004). No significant differences were found between the two groups in terms of sex and race mismatch between donor and recipient, nor in the donor-recipient ratio for weight or BMI.

**TABLE 2:** Donor characteristics and donor/recipient ratio.

Variable	Group NS N = 129	Group S N = 17	P
Comorbidities			
Arterial Hypertension	14 (11%)	2 (12%)	1.000
Diabetes Mellitus	5 (4%)	0 (0%)	0.962
Asthma	3 (2%)	0 (0%)	0.853
Smokers	28 (22%)	7 (41%)	0.121
Age ratio D/R	0.845 (0.25–2.37)	0.990 (0.50–2.04)	**0.041**
Weight Ratio D/R	1.00 (0.63–1.70)	0.995 (0.76–2.00)	0.527
Height Ratio D/R	1.01 (0.91–1.11)	1.04 (0.98–1.17)	**0.004**
BMI Ratio D/R	1.00 (0.57–1.73)	0.935 (0.67–1.73)	0.560
Sex mismatch	31 (24.0%)	5 (29.4%)	0.734
Race mismatch	17 (13.2%)	5 (29.4%)	0.111

The categorical variables are presented as percentages; the continuous variables are expressed as the median and interquartile range (IQR). D/R, Donor/Recipient; BMI, Body Mass Index. Significant p values are reported in bold type.

The correlation analysis showed that only the ratio between the donor’s and recipient’s height was considered a predictive factor for the need for graft sizing (p = 0.02, OR 2.70e + 10, 95% CI 41.4–1.87e + 19).

Table 3 illustrates the types of lung resections performed on the graft following the diagnosis of mismatch. The majority of patients (n = 11, 64.7%) underwent anatomical lung resections, most commonly involving the removal of two lobes/segments (n = 7, 41.2%). The most common anatomical resections performed were middle lobectomy (n = 9, 52.9%) and left lingular segmentectomy (n = 5, 29.4%).

**TABLE 3:** Types of graft resections performed for donor-recipient mismatch (every resection is reported separately, even multiple resection cases).

Type of resection	N	%
Atypical (wedges)	6	35.3%
Anatomical	11	64.7%
Segmentectomy		
Lingula	5	29.4%
Apex	2	11.8%
Lobectomy		
ML	9	52.9%
RUL	1	5.9%
RLL	1	5.9%
LUL	2	11.8%
LLL	1	5.9%
Bilobectomy	2	11.8%
Type of resection		
<1lobe/segment	6	35.3%
= 1 lobe/segment	2	11.8%
= 2 lobes/segments	7	41.2%
= 3 lobes/segments	2	11.8%

LLL, left lower lobectomy; LUL, left upper lobectomy; RML, right middle lobectomy; RLL, right lower lobectomy; RUL, right upper lobe.

Postoperative outcomes are summarized in Table 4. Although not statistically significant, a shorter ischemia time was observed in lungs that underwent sizing, both for the first lung (238 vs. 294 min in Group S and NS, respectively, p = 0.053) and the second lung (396 vs. 333 min in Groups S and NS, respectively, p = 0.108). Similarly, although not statistically significant, PGD3 was more frequent in Group S (53% vs. 29.7% in Group NS, p = 0.13). Although not statistically significant, a higher rate of prolonged mechanical ventilation (41.1% vs. 30.5% in Groups S and NS, respectively, p = 0.361), postoperative ECMO requirement (29.4% vs. 16.4% in Groups S and NS, respectively, p = 0.189), and acute kidney injury (29.4% vs. 21.1% in Groups S and NS, respectively, p = 0.196) was observed in patients who underwent lung resection. Additionally, it was noted that, starting from 3 months after the procedure, FEV1 decreased in patients who underwent graft resection, a phenomenon not observed in patients who did not undergo sizing, where FEV1 remained stable at 3 months post-operation, although this finding did not reach statistical significance (reduction of 0.04 mL and 0.35 mL in Groups NS and S, respectively, from 1st to 3rd month post-surgery, p = 0.077). No differences were observed in the length of hospital stay. The development of CLAD was lower, though not statistically significant, in Group S (23.5%, p = 0.382).

**TABLE 4:** Patients’ clinical outcomes after lung transplantation.

Outcomes	Group NS N = 128	Group S N = 17	P
First lung ischemia (min)	294 (108)	238 (86)	0.053
Second lung ischemia (min)	396 (144)	333 (141)	0.108
FEV1 1 month (mL)	2.18 (0.57–3.47)	2.10 (1.46–2.38)	0.487
FEV1 2 months (mL)	2.18 (1.13–3.36)	1.77 (1.08–2.86)	0.080
FEV1 3 months (mL)	2.14 (0.93–427)	1.75 (1.33–2.46)	0.077
PGD			0.130
Grade 1	23 (18.0%)	4 (23.5%)	
Grade 2	36 (28.1%)	3 (17.6%)	
Grade 3	38 (29.7%)	9 (53.0%)	
Acute Kidney Injury	27 (21.1%)	5 (29.4%)	0.196
Prolonged MV (>5 days)	39 (30.5%)	7 (41.1%)	0.361
Post-operative ECMO	21 (16.4%)	5 (29.4%)	0.189
CLAD	44 (34.4%)	4 (23.5%)	0.382
In-hospital stay (days)	37 (0–403)	34 (9–109)	0.583
OS (months)	36.8 (39.4)	25.2 (34.8)	0.098

Data are shown as medians with interquartile range (IQR) or absolute numbers with percentage when adequate. PGD: primary graft dysfunction; MV: mechanical ventilation; ECMO: extracorporeal membrane oxygenation; CLAD: chronic lung allograft dysfunction; OS: overall survival.

**FIGURE 1:**
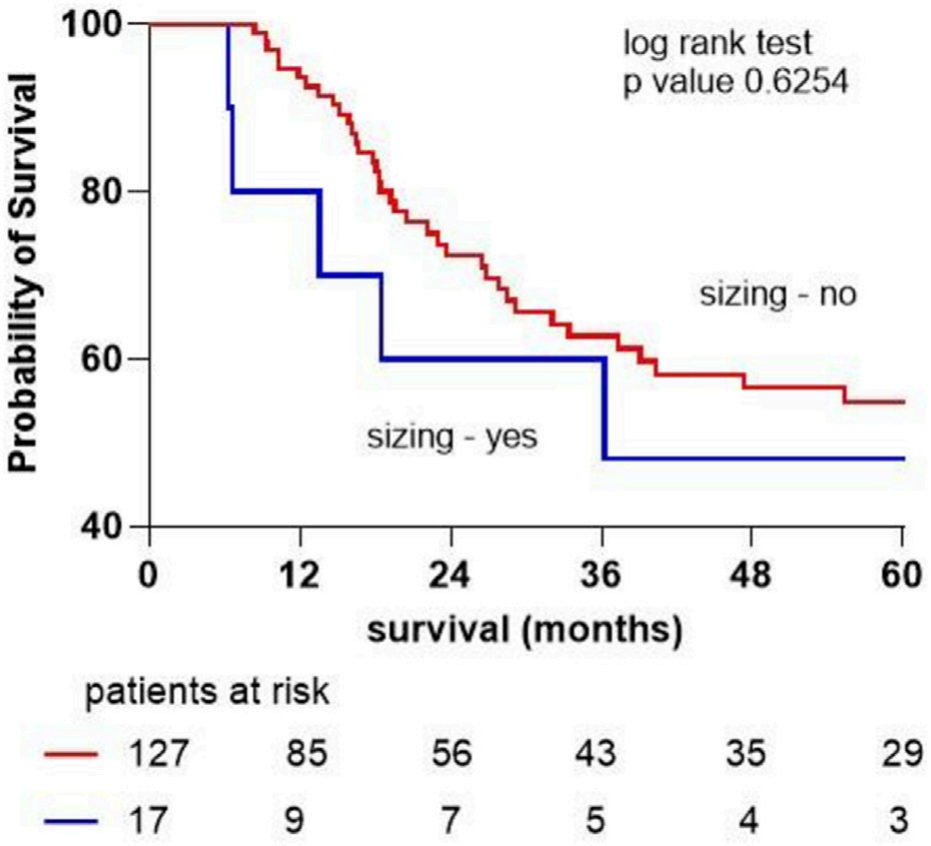
Kaplan-Meier survival curve for patients not undergoing lung resection and those undergoing graft sizing due to mismatch.

Survival analysis using Kaplan-Meier demonstrated no statistically significant difference in overall survival between the two groups (p = 0.625) (Figure 1).

To further investigate potential differences in outcomes within the group of patients undergoing lung graft sizing, stratification was performed based on age, sex, BMI, type of end-stage lung disease, and type of lung resection performed. The results of this stratification are presented in Table 5.

**TABLE 5:** Outcomes in the group of patients undergoing graft reduction, stratified by clinical characteristics and type of resection performed.

Subgroups	N	OS (months)	Degenza (days)	PGD	PGD grade 1	PGD grade 2	PGD grade 3	CLAD	AKI
BMI > 25	8	30 (35)	51 (33)	7 (84%)	1 (12%)	3 (36%)	3 (36%)	1 (12%)	3 (36%)
BMI < 25	9	19 (35)	31 (13)	9 (100%)	3 (33%)	0 (0%)	6 (67%)	3 (33%)	2 (22%)
p		0.423	0.289	0.114				0.312	0.293
Females	9	13 (16)	38 (30)	8 (89%)	1 (11%)	2 (22%)	5 (55%)	0 (0%)	0 (0%)
Males	8	39 (45)	46 (25)	8 (100%)	3 (36%)	1 (13%)	4 (50%)	4 (50%)	4 (50%)
p		0.277	0.413	0.494				**0.015**	**0.016**
Age < 50years	7	22 (37)	30 (14)	6 (86%)	1 (14%)	3 (42%)	2 (28%)	1 (14%)	4 (25%)
Age > 50years	10	27 (35)	50 (32)	10 (100%)	3 (30%)	0 (0%)	7 (70%)	3 (30%)	3 (60%)
p		0.601	0.241	**0.05**				0.452	0.293
Restrictive disease	8	16 (17)	48 (37)	7 (84%)	2 (25%)	0 (0%)	5 (75%)	1 (17%)	2 (12%)
Obstructive disease	7	23 (43)	39 (16)	7 (100%)	1 (28%)	1 (28%)	4 (66%)	1 (13%)	2 (12%)
p		0.754	0.846	0.391				0.825	1.000
Atypical resection	6	41 (38)	34 (15)	5 (83%)	2 (33%)	0 (0%)	3 (50%)	3 (50%)	0 (0%)
Anatomical resection	11	16 (31)	46 (32)	11 (100%)	2 (18%)	3 (27%)	6 (54%)	1 (9%)	5 (45%)
p		0.098	0.615	0.277				0.057	**0.018**

Data are shown as medians with interquartile range (IQR) or percentage when adequate. AKI, Acute Kindney Injury; CLAD, Chronic Lung Allograft Dysfunction; PGD, Primary Graft Dysfunction; OS, Overall Survival. Significant p values are reported in bold type.

Within the Sizing Group, there were no differences in outcomes based on the recipient’s BMI or the underlying type of lung disease. In the male subgroup undergoing sizing, a higher rate of CLAD onset was observed (4 patients, 50% in the male group, none in the female group, p = 0.015) as well as AKI (4 patients, 50% in the male group, none in the female group, p = 0.016). In patients over 50 years old, a higher rate of PGD was observed (100% in the > 50 years group vs. 86% in the < 50 years group). In the group undergoing anatomical lung resection, the onset of AKI was statistically significant (45% in the anatomical resection group vs. 0% in the non-anatomical resection group, p = 0.018). Although not statistically significant, PGD development was observed in all patients with a BMI < 25, all male patients, all patients over 50 years old, those with obstructive lung disease, and all patients undergoing anatomical resection.

## Discussion

Lung transplantation is a life-saving intervention for patients with end-stage lung disease, but donor-recipient mismatch, particularly in terms of lung size, can contribute to significant postoperative complications. These complications, including PGD, bronchiolitis obliterans syndrome, and overall reduced graft survival, highlight the importance of optimizing donor-recipient matching.

Our results indicate that donor-recipient mismatch, particularly in terms of donor-recipient height, plays a crucial role in determining the necessity for graft sizing. Specifically, a height discrepancy between donor and recipient was significant between the two groups, with shorter recipients in the Sizing Group (p = 0.004). This is consistent with previous studies [5] suggesting that lung size and thoracic dimensions are critical factors in ensuring a functional match between donor lungs and recipients.

Another significant indicator of mismatch was the donor-recipient age ratio. The results show that the ratio was 0.99 in the Sizing Group, compared to a lower ratio (0.845) in the Non-Sizing Group. This result may indicate a bias in organ allocation based on donor age, where, for ethical reasons, younger organs are preferentially allocated to younger recipients, and older organs to older recipients. This ethical factor may sometimes take precedence over D/R size matching, which could contribute to mismatches and the need for sizing.

The only predictive factor for the need for graft sizing was the ratio between donor and recipient height (p = 0.02). Interestingly, factors such as BMI, weight, and sex did not appear to predict the necessity for graft resizing, further emphasizing the importance of anatomical dimensions, such as height, over overall body mass in determining compatibility, in contrast to what has been observed in cardiac transplantation [14, 15].

A statistically significant difference was observed in the type of transplant performed, with a greater need for sizing in bilateral transplants (p = 0.02), as previously noted in other studies [16]. Single lung transplantation, especially in cases of pulmonary fibrosis or COPD, can lead to adaptation of the intrathoracic structures, with the graft tending to overinflate and occupy more space, creating an intrathoracic asymmetry between the native lung and the transplanted lung [17]. Due to the possible deviation of the structures toward the native lung, a larger donor lung can be used in a single transplant without the need for sizing. This is not the case in bilateral transplants, where, in the event of oversizing, graft reduction is necessary.

Although not statistically significant, it is evident that the restrictive pattern is the most frequently represented among patients who underwent sizing. This suggests that the thoracic dimensions of patients with restrictive lung disease tend to overestimate the actual intrathoracic size, which more frequently leads to the need for graft trimming.

Another important observation is that patients who underwent graft sizing had higher Lung Allocation Scores (LAS) compared to those in the non-sizing group (31 vs. 20). This difference, although not statistically significant, likely reflects the urgency under which these transplants were conducted. In many cases, organs that were less dimensionally compatible were allocated to patients with more critical conditions and higher LAS. This emphasizes the complex decision-making process in organ allocation, particularly in urgent transplant situations, where matching is often secondary to the need for a life-saving procedure [18]. This observation may also explain the lower overall survival (OS) in the Sizing Group compared to the NS Group, although not statistically significant (36.8 vs. 25.2 months in Group NS and Group S, respectively, p = 0.625), as patients in the Sizing Group had a higher mortality risk for their critical conditions. The numerical trend toward lower survival in the Sizing Group (25.2 vs. 36.8 months) suggests a need for longer follow-up studies to assess the long-term impact of graft resizing on survival and CLAD.

In our study, the most common surgical approach to address dimensional mismatch was anatomical resections, most commonly middle lobectomy and lingulectomy, similar to other studies [19]. This targeted approach suggests that the most common mismatch is related to the lung’s shape and volume in the antero-posterior direction, which necessitates the removal of portions of the middle or lingular lobes. Furthermore, the majority of graft sizings involved the removal of two segments/lobes, indicating a bilateral dimensional mismatch.

In the study, there were no statistically significant differences in postoperative outcomes between the sizing and non-sizing groups, suggesting that lung resection remains a viable option in cases of donor-recipient size mismatch, especially in situations of donor scarcity. As observed, a reduced recipient height can contribute to increased waiting times and a higher risk of mortality on the waiting list [20]. Nevertheless, a trend toward worse outcomes, such as higher rates of PGD3 (29.7% vs. 53.0% in Groups NS and S, respectively) and extended mechanical ventilation (30.5% vs. 41.1% in Groups NS and S, respectively), was observed in the sizing group. This suggests that graft resizing may be associated with more complex procedures and potentially poorer short-term outcomes. Therefore, although it is a procedure that expands the donor pool, a careful clinical assessment is needed to ensure the best treatment for each individual recipient.

The correlation between graft reduction and a higher rate of PGD3 is likely due to three factors. First, when a graft is resized, particularly through anatomical resections, the vascular bed of the donor lung is reduced. This reduction alters the distribution of blood flow to the remaining lung tissue. After resection, blood flow to the remaining lung segments may increase to compensate for the reduced surface area, potentially leading to capillary-alveolar damage and the leakage of fluid into the alveolar spaces. This vascular redistribution can exacerbate PGD, as impaired gas exchange occurs due to the accumulation of fluid and damage to the alveolar-capillary membrane. Secondly, undersized grafts, particularly when they are overinflated to fit within the recipient’s thoracic cavity, pose a significant risk for mechanical ventilation injury. Overinflation leads to ventilator-induced lung injury, as excessive tidal volumes and pressures can damage the alveolar walls and exacerbate PGD. This is a known phenomenon in mechanical ventilation, particularly when the lung is artificially expanded beyond its optimal volume. Additionally, hyperinflation in undersized grafts increases the risk of barotrauma, contributing to ventilator-induced damage, which may lead to prolonged mechanical ventilation and poorer overall outcomes. [21–23]. Finally, another mechanism resulting from graft resizing is the increased risk of pulmonary edema. In cases where significant lung tissue is removed to match the donor and recipient size, the remaining lung tissue may be more susceptible to fluid buildup. The reduction in lung volume can lead to impaired lymphatic drainage and increased capillary permeability, particularly in the post-operative period. This results in pulmonary edema, further impairing gas exchange and contributing to the development of PGD and CLAD over time.

While the study found no significant differences in survival between the groups, it is worth noting the potential long-term impact of graft sizing on overall graft function. The FEV1 values, although not statistically significant, were lower in the graft-sizing group after 3 months, suggesting that the initial postoperative challenges may extend into longer-term pulmonary function. These findings align with previous studies, which have shown that lung size mismatch can negatively impact graft function, especially in bilateral lung transplantation [24].

One notable aspect of our study is the stratification analysis, which revealed that factors such as male sex, age over 50 years, and anatomical sizing (such as segmentectomy or lobectomy) may influence the occurrence of specific complications like PGD, acute kidney injury (AKI), and CLAD. The relevance of this observation lies in the fact that older patients typically have diminished physiological reserves, which may exacerbate the effects of graft sizing. Advanced age is a recognized risk factor for increased mortality and complications after lung transplantation, possibly due to age-related changes in pulmonary and systemic vascular function, immune response, and wound healing. Older males, in particular, may face compounded risks due to gender-specific differences in immune response, which can influence both graft rejection and long-term survival. Given these factors, it would be necessary to consider tailored monitoring strategies for high-risk subgroups, including adjusting immunosuppressive therapy, managing fluid balance carefully to avoid AKI, and using advanced ventilation strategies to minimize mechanical damage to the lung. By identifying these at-risk populations early and adjusting perioperative management accordingly, it may be possible to reduce complications and improve overall outcomes.

Additionally, special attention should be paid to the development of PGD in all patients, particularly those who are male, over 50 years old, have obstructive lung disease, and undergo anatomical resections. As observed in our study and supported by the literature, anatomical resections (lobectomy or segmentectomy) are more commonly associated with complications such as AKI and PGD. These resections involve removing larger portions of lung tissue, which leads to more significant changes in the vascular bed and mechanical function of the graft. In contrast, preserving more lung tissue, atypical resections may mitigate the extent of vascular disruption, reducing the likelihood of pulmonary edema and mechanical ventilation injury [6, 25].

The higher incidence of CLAD in male patients may be related to the development of PGD, which is now recognized as a risk factor for the development of CLAD [26]. The reduction in the vascular bed, combined with an increased risk of pulmonary edema, also necessitates maintaining a more negative electrolyte balance and the use of vasoconstrictors in patients undergoing graft sizing. This can lead, especially in older patients, to the development of AKI in the postoperative period due to renal hypoperfusion. This complication was found to be significant not only in male patients but also in those who underwent anatomical resections.

These results highlight the importance of anticipating the need for graft sizing and carefully assessing the individual recipient’s risk of requiring sizing. Based on the observations, graft volume reduction is likely preferable in female recipients, those under 50 years old, and those with restrictive lung disease. Furthermore, the role of atypical resections in cases of mismatch should certainly be reevaluated in comparison to anatomical resections.

### Limitations of the Study

This study has several limitations that should be considered. Firstly, it is a retrospective analysis, which inherently limits the ability to establish causal relationships and may introduce selection bias. The relatively small sample size, especially in the Sizing Group (n = 17), may limit the statistical power to detect significant differences in complications. Multi-center studies are needed to validate these findings and refine clinical guidelines for graft resizing. Another limitation is the absence of a standardized protocol for graft resizing, as surgical decisions were based on clinical judgment, which may introduce variability in the outcomes. Moreover, a potential bias of the study lies in the fact that the immunosuppressive therapy of the patients and whether the donor lungs were standard or marginal were not considered, which could clearly influence the outcomes observed. In summary, while the study provides valuable insights, further research with a larger, prospective cohort and longer follow-up is needed to validate these findings and refine the criteria for graft resizing in lung transplantation.

## Conclusion

Height discrepancy between donor and recipient is a key predictor for resizing, aligning with previous research emphasizing the importance of anatomical dimensions over other factors like BMI or sex. Although graft resizing is a viable solution for size mismatches, it may be associated with worse short-term outcomes, such as higher rates of PGD and prolonged mechanical ventilation, especially in patients with obstructive pulmonary disease, older males, and those undergoing anatomical resection. These findings emphasize the importance of preoperative donor-recipient size matching, particularly in male recipients over 50 and those with obstructive lung disease. When resizing is unavoidable, non-anatomical resections may be preferred to minimize postoperative complications.

## Data Availability

The raw data supporting the conclusions of this article will be made available by the authors, without undue reservation.
